# Bergenin protects pancreatic beta cells against cytokine-induced apoptosis in INS-1E cells

**DOI:** 10.1371/journal.pone.0241349

**Published:** 2020-12-21

**Authors:** Sajid Ali Rajput, Munazza Raza Mirza, M. Iqbal Choudhary

**Affiliations:** 1 Dr. Panjwani Center for Molecular Medicine and Drug Research, International Center of Chemical and Biological Sciences, University of Karachi, Karachi, Sindh, Pakistan; 2 H. E. J. Research Institute of Chemistry, International Center for Chemical and Biological Sciences, University of Karachi, Karachi, Sindh, Pakistan; 3 Department of Biochemistry, Faculty of Science, King Abdulaziz University, Jeddah, Saudi Arabia; Broad Institute, UNITED STATES

## Abstract

Beta cell apoptosis induced by proinflammatory cytokines is one of the hallmarks of diabetes. Small molecules which can inhibit the cytokine-induced apoptosis could lead to new drug candidates that can be used in combination with existing therapeutic interventions against diabetes. The current study evaluated several effects of bergenin, an isocoumarin derivative, in beta cells in the presence of cytokines. These included (i) increase in beta cell viability (by measuring cellular ATP levels) (ii) suppression of beta cell apoptosis (by measuring caspase activity), (iii) improvement in beta cell function (by measuring glucose-stimulated insulin secretion), and (iv) improvement of beta cells mitochondrial physiological functions. The experiments were carried out using rat beta INS-1E cell line in the presence or absence of bergenin and a cocktail of proinflammatory cytokines (interleukin-1beta, tumor necrosis factor-alpha, and interferon- gamma) for 48 hr. Bergenin significantly inhibited beta cell apoptosis, as inferred from the reduction in the caspase-3 activity (IC_50_ = 7.29 ± 2.45 μM), and concurrently increased cellular ATP Levels (EC_50_ = 1.97 ± 0.47 μM). Bergenin also significantly enhanced insulin secretion (EC_50_ = 6.73 ± 2.15 μM) in INS-1E cells, presumably because of the decreased nitric oxide production (IC_50_ = 6.82 ± 2.83 μM). Bergenin restored mitochondrial membrane potential (EC_50_ = 2.27 ± 0.83 μM), decreased ROS production (IC_50_ = 14.63 ± 3.18 μM), and improved mitochondrial dehydrogenase activity (EC_50_ = 1.39 ± 0.62 μM). This study shows for the first time that bergenin protected beta cells from cytokine-induced apoptosis and restored insulin secretory function by virtue of its anti-inflammatory, antioxidant and anti-apoptotic properties. To sum up, the above mentioned data highlight bergenin as a promising anti-apoptotic agent in the context of diabetes.

## Introduction

Medicinal plants contain a wide variety of pharmacologically important bioactive compounds, such as flavonoids, quinines, tannins, and ascorbic acid reported for their antioxidant, anti-inflammatory, and hypoglycemic properties. The plants of *Begenia* genus have traditionally been used for the treatment of diarrhea, cough, ulcer, vomiting, and kidney stones [1, 2]. The extracts of *Bergenia* rhizomes have also been reported for their anti-inflammatory, analgesic, antibacterial, and diuretic properties. Moreover, these extracts have also been topically applied to the wounds, eyesores, and boils [3–5]. Bergenin, a *C*-glucoside of 4-*O*-methylgallic acid, is naturally found in the rhizomes of *Caesalpinia digyna*, *Mallotus japonicas*, *Mallotus philippinensis*, *Corylopsis spicata*, *Sacoglottis gabonensis*, and *Bergenial crassifolia*. Other components obtained from *Bergenia* species include bergenan, β-sitosterol, polyphenols, and galloylarbutin. Bergenin, an isocoumarin derivative with five hydoxyl groups, is reported several important pharmacological activities, such as anti-inflammatory, hypolipidimic, antimalarial, hepatoprotective, antiarrhythmic, anti-HIV, and neuroprotective activities [6–10]. The hepatoprotective, and neuroprotective activities of bergenin were reported to be mediated through its free radical scavenging property in both *in vitro*, and *in vivo* models [11]. Our previous study had shown anti-inflammatory properties of bergenin, where it inhibited the production of inflammatory mediators, such as NO, and TNF-α [12].

Diabetes mellitus has reached an epidemic proportion globally, with 463 million people currently suffering from this disease according to the International Diabetes Federation [13]. Diabetes patients exhibit persistent hyperglycemia due to the impairment of beta cell insulin secretory function, insulin action or both [14, 15]. Pancreatic beta cells are reported to have low antioxidant potential, and are sensitive towards reactive oxygen (ROS), and reactive nitrogen species (RNS). This oxidative stress may ultimately lead to the impairment in beta cell insulin secretory function. In both type1, and type 2 diabetes, beta cells mass is significantly reduced due to apoptosis. The loss of beta cell identity is also reported to be one of the hallmarks of reduced functional beta cell mass [16]. At early stages of diabetes, interleukin-1β (IL-1β) induces the intrinsic apoptotic pathway in beta cells that eventually results in hyperglycemic condition in the diabetic patients. As such, at the time of disease diagnosis, beta cell population is reported to be decreased by 70–80% in type 1 diabetes, and about 50% in type 2 diabetes patients [17–20].

Proinflammatory cytokines play prominent roles in beta cell dysfunction, and death. Interleukin-1β (IL-1β,) interferon-γ (INF-γ), and tumor necrosis factor-α (TNF-α) have been employed in *in vitro* studies to mimic the situations which induce pancreatic beta cell death. Moreover, these cytokines have been shown to stimulate JAK-STAT, and NFκB pathways, which later induce intrinsic apoptotic pathway in beta cells. Likewise, both TNF-α, and IL-1β have also been reported to induce nitric oxide (NO) production, which causes the inhibition of electron transport chain, decrease in glucose oxidation rate leading to decrease in ATP generation, and insulin production [21–23]. Therapeutic interventions currently available to treat diabetes are unable to cease the loss of functional beta cell mass. Therefore, strategies targeting beta cell apoptosis are urgently required. As such, small molecule compounds inhibiting cytokine-induced pancreatic beta cell death, could serve as new drug candidates that may be used in combination with existing therapeutics.

The hypothesis of this study was that bergenin can protect beta cells from cytokine-induced apoptosis and can restore beta cell insulin secretory function. To demonstrate this, we employed cell-based assays and examined the effects of bergenin in two-day treatment of INS-1E cells with a cytokine cocktail (IL-1β, IFN-γ, and TNF-α). Using this strategy, we demonstrated that bergenin prevented cytokine-induced beta cell apoptosis, and at the same time, restored glucose-stimulated insulin secretion. Bergenin protected beta cells from the adverse effects of proinflammtory cytokines by virtue of its anti-inflammatory, antioxidant, and antiapoptotic properties.

## Materials and methods

### Cell culture and reagents

INS-1E is a derivative of INS-1 cells originally established from an x-ray induced insulinoma in rat [24]. INS-1E cell line was generously provided by Paolo Meda, Department of Morphology, University of Geneva Medical School, Switzerland. The INS-1E cells were negative for mycoplasma contamination, and were verified by the Venor^™^ GeM Mycoplasma Detection Kit (Sigma, St. Louis, USA). INS-1E cells were cultivated in complete medium composed of RPMI 1640 medium supplemented with 11 mM glucose, glutamine, 10% fetal bovine serum, 50 IU/mL penicillin, 50 mg/L streptomycin, 10 mM HEPES, 50 μM beta-mercaptoethanol, and 1 mM sodium pyruvate. INS-1E cells were grown in a 37°C incubator with 5% CO_2_ in a humidified atmosphere, and were split every week. All cell culture reagents and supplements were obtained from Gibco (Sigma, St. Louis, USA). Whereas, recombinant rat IL-1β, mouse IFN-γ, and mouse TNF-α were purchased from R&D Systems (Minneapolis, MN, USA). Cell Titer-Glo and caspase-Glo 3/7 reagents were purchased from Promega (Fitchburg, Wisconsin, USA). Ultrasensitive rat insulin ELISA kit was purchased from ALPCO (Salem, NH, USA). Alexa Fluor® 488 Annexin V/Dead Cell Apoptosis kit was purchased from Molecular Probes (Invitrogen, Waltham, USA). Bergenin and Griess reagent were purchased from Sigma, St. Louis, USA.

### Measurement of the cellular ATP levels

**Fig 1:**
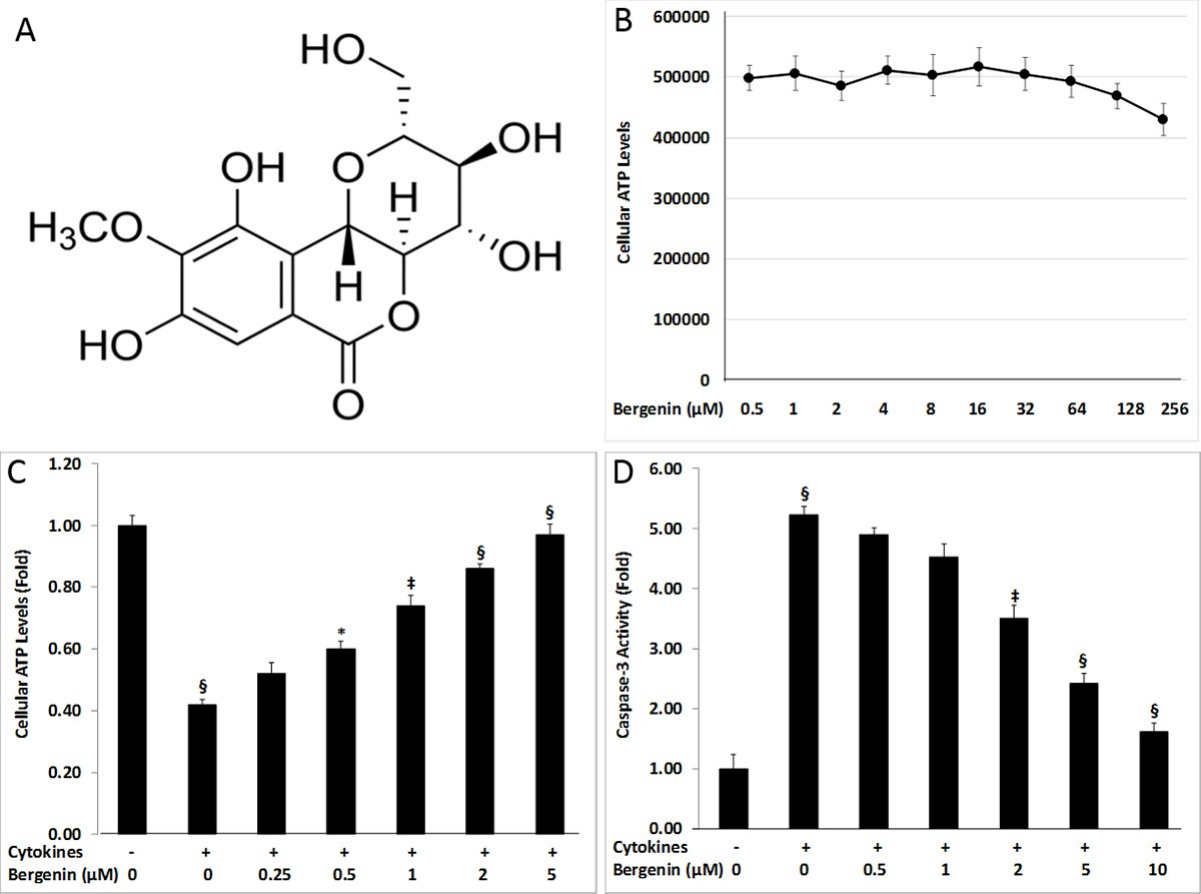
Suppression of cytokine-induced beta cell damage by bergenin. (A) The chemical structure of bergenin. (B) Cellular ATP levels of INS-1E cells treated for two days with bergenin in the absence of cytokine treatment. INS-1E cells were treated for two days with proinflammatory cytokines (IL-1β, INF-γ, and TNF-α) in the presence or absence of bergenin (0.25–10 μM). (C) Dose-dependent effects of bergenin on cellular ATP levels after 48 hr treatment with cytokines. (D) Dose-dependent effects of bergenin on caspase-3 activity after 48 hr of treatment with cytokines. Data are represented as the mean ± standard deviation of 12 independent wells for A-B. * indicates p <0.05, ‡<0.01, and §<0.001 relative to cytokine-treated cells.

We employed quantitation of cellular ATP levels as our primary assay, which served as a surrogate marker for beta cell viability. The CellTiter-Glo luminescent cell viability assay (Promega, Madison, WI, USA) was used to quantify cellular ATP levels in metabolically active cells, which indirectly determine the number of viable cells in the experimental culture medium. Ribonucleotide adenosine triphosphates (rATPs) (Promega) were used for the standard curve. In this study, we prepared a cytokine cocktail (20 ng/mL IL-1β, 50 ng/mL, IFN-γ, and 50 ng/mL TNF-α) to model the events leading to beta cell death as previously described [25]. INS-1E cells were seeded at 3 x 10^4^ cells per well in white optical 96-well plates (Perkin Elmer, USA). After overnight incubation, the exhausted RPMI medium was removed, and 150 μL of fresh RPMI medium containing a cytokine cocktail was added to each well. Bergenin (Fig 1F) was dissolved in DMSO in required dilution. We used 10 μM as a common bergenin concentration in the assays. For dose-response studies, INS-1E cells were treated with bergenin at concentrations from 0.5 to 20 μM. A stock solution of bergenin was diluted in the culture medium. DMSO was added in the medium without bergenin treatment as a vehicle control (these steps were similar for other cell based assays). After 48 hr incubation at 37°C, the RPMI medium was removed, and 50 μL of CellTiter-Glo reagent was added. After 10 min of incubation at room temperature, the luminescence was measured using SpectraMax 5M^e^ microplate reader (Molecular Devices, CA, USA).

### Caspase-3 activity assay

For the analysis of caspase-3 activity, INS-1E cells were incubated for two days as described for cellular ATP levels assay. After treatment with cytokines and bergenin for 48 hr, 50 μL of Caspase-Glo 3/7 reagent was added to each well. After 2 hr incubation at room temperature, luminescence was measured using SpectraMax 5M^e^ microplate reader (Molecular Devices, USA).

### Measurement of the cellular nitrite production

For the measurement of cellular nitric oxide production, INS-1E cells were incubated for two days as described for cellular ATP levels assay. After treatment with cytokines and bergenin for 48 hr, 100 μL modified Griess reagent (1:1 mixture of 1% sulfanilamide in 30% acetic acid and 0.1% *N*-(1-naphthyl) ethylenediamine dihydrochloride in 60% acetic acid) was added to each well. After 5 min of incubation at room temperature, the absorbance was measured at 540 nm using SpectraMax 5M^e^ microplate reader (Molecular Devices, USA).

### Measurement of the mitochondrial membrane potential

For the measurement of mitochondrial membrane potential, INS-1E cells were incubated for two days as described for cellular ATP levels assay. After treatment with cytokines and bergenin for 48 hr, 20 μL of 3.25 mM JC-1 was added to each well. After 3 hr incubation at 37°C, the cells were gently washed three times with 150 μL of 1X calcium- and magnesium-free PBS. Fluorescence was measured with SpectraMax 5M^e^ microplate reader (Molecular Devices, USA) at the rhodamine spectra (excitation/emission 530 nm/580 nm), followed by fluorescein (excitation/emission 485 nm/530 nm). The ratio of rhodamine to fluorescein intensity was determined which represents the degree of mitochondrial membrane potential.

### Measurement of glucose-stimulated insulin secretion (GSIS)

For the quantitation of glucose-stimulated insulin secretion in beta cells, INS-1E cells were seeded in 24-well plates at 5 x 10^4^ cells per well (Perkin Elmer, USA). After overnight incubation, 150 μL of fresh RPMI medium containing a combination of cytokines (20 ng/mL IL-1β, 50 ng/mL, IFN-γ, and 50 ng/mL TNF-α) was added to each well. For dose-response studies, bergenin was added at (1–10 μM) concentrations. After 48 hr incubation, INS-1E cells were washed twice with 500 μL glucose-free Krebs-Ringer Bicarbonate buffer (KRB) (118 mM NaCl, 4.8 mM KCl, 1.2 mM NaH_2_PO_4_·2(H_2_O), 1.0 mM MgSO_4_·7(H_2_O), 25 mM NaHCO_3_, and 2.4 mM CaCl_2_·2(H_2_O). Thereafter, INS-1E cells were incubated in 500 μL of pre-warmed KRB under low (2 mM) and high (16 mM) glucose for 1 hr. The supernatant was taken in a new tube for the measurement of released insulin. To extract cellular insulin content, INS-1E were washed twice with ice-cold PBS (with Mg^2+^ and Ca^2+^), followed by the addition of 500 μL of lysis buffer (1% Triton X-100, 20 mM HEPES pH 7.9, 0.3 mM NaCl, 1.5 mM MgCl_2_, 0.2 mM EDTA, cOmplete Mini Protease Inhibitor Cocktail and PhosStop (Roche, Germany). After three freeze/thaw cycles (−80°C /4°C), INS-1E cells were centrifuged for 20 sec. at 6000*g at room temperature, and supernatant was collected in a new tube for the quantitation of cellular insulin content. We compared the percent of insulin secreted, relative to the insulin content in each well. Insulin was measured using the ultra-sensitive rat insulin ELISA kit (Alpco Diagnostics, Salem, USA).

### Determination of the intracellular ROS production

The cellular ROS production was measured using fluorescent probe CM-H_2_DCFDA (Invitrogen, USA). INS-1E cells were seeded at 3 x 10^4^ cells per well in 96-well black fluorescence plates (Perkin Elmer, USA). After overnight incubation with 10 μM fluorescent probe CM-H_2_DCFDA, 150 μL of RPMI containing bergenin, and cytokine cocktail were added to each well for 48 hr. After incubation, fluorescence of the treated cells was measured (which corresponded to the intracellular ROS) using a fluorescence spectrophotometer (SpectraMax 5M^e^; Molecular Devices, USA) at excitation, and emission wavelengths of 485 and 530 nm, respectively.

The ROS levels were calculated using the formula: % Inhibition = 100 –[(Fluorescence of test compound–Fluorescence of blank) / (Fluorescence of control–Fluorescence of blank) x 100].

### Measurement of mitochondrial dehydrogenase activity

The mitochondrial dehydrogenase activity was measured using the MTT colorimetric assay. INS-1E cells were incubated for two days as described for cellular ATP levels assay. After 48 hr incubation with cytokine cocktail and bergenin, cells were observed under a contrast phase microscope before adding freshly prepared MTT solution. The medium was replaced with 200 μL of MTT, and the plates were incubated for 4 hr at 37°C. The dark blue formazan crystals were formed in the intact cells that were solubilized using 100 μL of DMSO. Absorbance was measured at 540 nm using SpectraMax 5M^e^ microplate reader (Molecular Devices, USA).

### No cytokine measurement of the cellular ATP levels

A counter screen assay was performed to test the ability of bergenin for the induction of ATP production in the absence of cytokines. INS-1E cells were treated with bergenin at various doses for 48 hr in the absence of cytokine cocktail. INS-1E cells were seeded at 3 x 10^4^ cells per well in an opaque, white 96-well plates (Perkin Elmer, USA) in 150 μL of RPMI containing bergenin for two days. After 48 hr incubation, 50 μL of CellTiter-Glo reagent was added, and incubated for 10 min at room temperature. Luminescence was measured using SpectraMax 5M^e^ microplate reader (Molecular Devices, USA).

### Flow cytometry analysis of beta cell apoptosis

Annexin V serves as a useful marker for the identification of apoptotic cells, since it binds to phosphatidylserine with high affinity in the presence of Ca^+2^ whereas, propidium iodide (PI) intercalates with DNA/RNA of non-viable cells due to lack of membrane integrity. Annexin V-FITC + propidium iodide staining works as follows: the viable cells are negative to both the annexin V/ PI dyes, whereas the apoptotic cells are stained with annexin V-FITC only. Moreover, the cells that take up PI are considered as dead cells; while those positive for both the annexin V/ PI dyes are considered as late apoptotic cells [26, 27].

INS-1E cells were seeded in 24-well plate (Perkin Elmer, USA) at a concentration of 3 x 10^6^ cells per well in 200 μL RPMI containing 5% FBS and incubated overnight at 5% CO_2_ at 37°C. The following day, the oxidized medium was replaced with the fresh one, and INS-1E cells were treated for 48 hr with a cytokine cocktail (20 ng/mL IL-1β, 50 ng/mL IFN-γ, and 50 ng/mL TNF-α), and bergenin at various concentrations (2–10 μM). The vehicle (DMSO), and positive control (no cytokines) wells were also used in the experiment. After incubation, the INS-1E cells were harvested by trypsinization, and washed twice with 1xPBS followed by centrifugation at 2,500 rpm at 4°C for 10 min. The cell pellet was then suspended in 1 mL of 1x binding buffer (annexin V-FITC/ PI buffer). From this solution, 100 μL of suspension (1 x 10^5^ cells) was then transferred to the Eppendorf tubes. We then added 5 μL of fluorescein isothiocianate (FITC) conjugated annexin-V molecules, and 5 μL of propidium iodide (PI = 50 μg/mL) and incubated for 15 minutes in the dark. After incubation, 200 μL of 1X Annexin binding buffer was added in the tube, and mixed gently. The samples were then proceeded for apoptotic analysis on FACS Caliber flow cytometer (USA). Propidium iodide with excitation/emission of 493/636 nm was detected on FL2 channel, while annexin V-FITC with excitation/emission of 494/518 nm was detected on FL1 channel. The analysis was carried out using Cell Quest Pro software. The viable cell populations were in the lower left quadrant (Annexin V^-^/PI^-^), the cells at the early apoptosis were in the lower right quadrant (Annexin V^+^/PI^-^), and the ones at the late apoptosis were in the upper right quadrant (Annexin V^+^/PI^+^). INS-1E cells in each quadrant were expressed as percentage (%) of the total number of stained cells counted.

### Statistical analysis

The EZ-Fit enzyme kinetics was used to calculate the EC_50_ and IC_50_ values. All data were derived from three to twelve independent experiments and presented as mean ± standard deviation (SD). Statistical significance was analyzed by Student-t test, and one-way ANOVA with Tukey’s post hoc test. The p-value of less than <0.05 was considered statistically significant, and indicated with *, while p-value <0.01, and <0.001 are marked with **‡** and **§**, respectively.

## Results

### Bergenin enhanced beta cell viability in the presence of cytokines

Bergenin is an isocoumarin derivative with five hydoxyl groups (Fig 1A). In order to quantify the maximum tolerable concentration of bergenin in experimental assays, INS-1E cells were cultured for 48 hr with bergenin at several concentrations in the absence of cytokines (Fig 1B). These values were used to determine the maximum tolerable concentration of bergenin in the follow-up assays. In beta cell viability assay, two-day treatment of INS-1E cells with a cytokine cocktail (IL-1β, INF-γ, and TNF-α) resulted in ˃2-fold decrease in cellular ATP levels as compared to untreated controls (Fig 1C). Bergenin completely restored beta cell ATP levels in a dose-dependent manner. Beta cell ATP levels were increased to ˃90% relative to untreated controls by 5 μM bergenin (Fig 1C).

### Bergenin caused inhibition of cytokine-induced caspase-3 activity

Cytokine-induced apoptosis was assessed by evaluating caspase-3 activity, which is a downstream effector of the apoptotic pathway. Two-day treatment of INS-1E cells with a cytokine cocktail resulted in almost 5-fold increase in caspase-3 activity as compared to the no cytokine-treated cells (Fig 1D); however, this increase in caspase activity was suppressed more than 80% by 10 μM bergenin (Fig 1D). Bergenin also reduced caspase-3 activity in a dose-dependent manner (Fig 1D). This assay demonstrated the role of bergenin in preventing beta cell apoptosis, and subsequent increase in beta cell viability.

### Bergenin inhibited cytokine-induced cellular nitrite production

**Fig 2:**
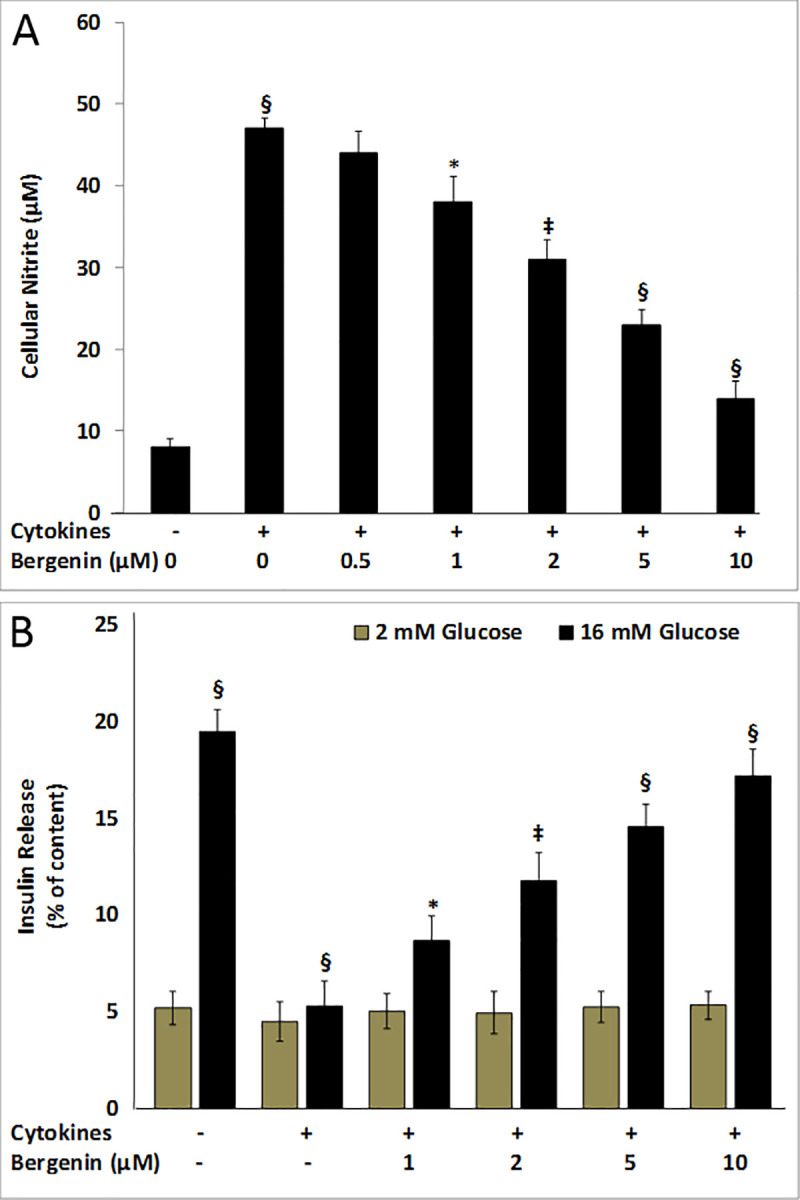
Inhibition of cellular nitrite production and restoration of glucose-stimulated insulin secretion. INS-1E cells were treated for two days with proinflammatory cytokines (IL-1β, INF-γ, and TNF-α) in the presence or absence of bergenin (0.25–10 μM). (A) Dose-dependent effects of bergenin on cellular nitrite production after 48 hr treatment with cytokines. Data are represented as the mean ± standard deviation of 12 independent wells for nitrite production. (B) The glucose-stimulated insulin secretion was measured in “low glucose” (2 mM), and “high glucose” (16 mM) conditions in the presence of bergenin. Data are represented as the mean ± standard deviation of six independent wells for insulin secretion. * indicates p <0.05, ‡<0.01, and §<0.001 relative to cytokine-treated cells.

Nitric oxide is very reactive molecule, whose production is stimulated by IL-1β, and IFN-γ, leading to apoptotic beta cell death. Cellular nitrite production, a stable oxidized product of nitric oxide, served as a surrogate marker for the NO production in INS-1E cells. Two-day treatment of INS-1E cells with a cytokine cocktail resulted in approximately 5-fold increase in the NO levels as compared to the untreated controls (Fig 2A). Bergenin was found to be effective in reducing the cellular nitrite production in a dose-dependent manner. Beta cells NO levels was decreased to almost 70% by 5 μM bergenin (Fig 2A).

### Bergenin restored the glucose-stimulated insulin secretion (GSIS)

We examined the effects of bergenin on GSIS in INS-1E cells. Under normal condition, stimulation with high-glucose (16 mM) resulted in approximately 4-fold increase in insulin secretion relative to low-glucose (2 mM) condition (Fig 2B). We compared the percent of insulin secreted, relative to the insulin content in each well. Two-day treatment of INS-1E cells with a cytokine cocktail reduced GSIS to ˃3.5-fold as compared to the untreated controls (Fig 2B); however, this loss of insulin secretion was significantly suppressed by the addition of 10 μM bergenin to INS-1E cells, with insulin secretion was elevated to ˃80% relative to cytokine-treated cells (Fig 2B). Bergenin also restored GSIS in a dose-dependent manner where insulin stimulation was enhanced to ˃3-fold relative to cytokine-treated cells (Fig 2B).

### Bergenin restored mitochondrial membrane potential

**Fig 3:**
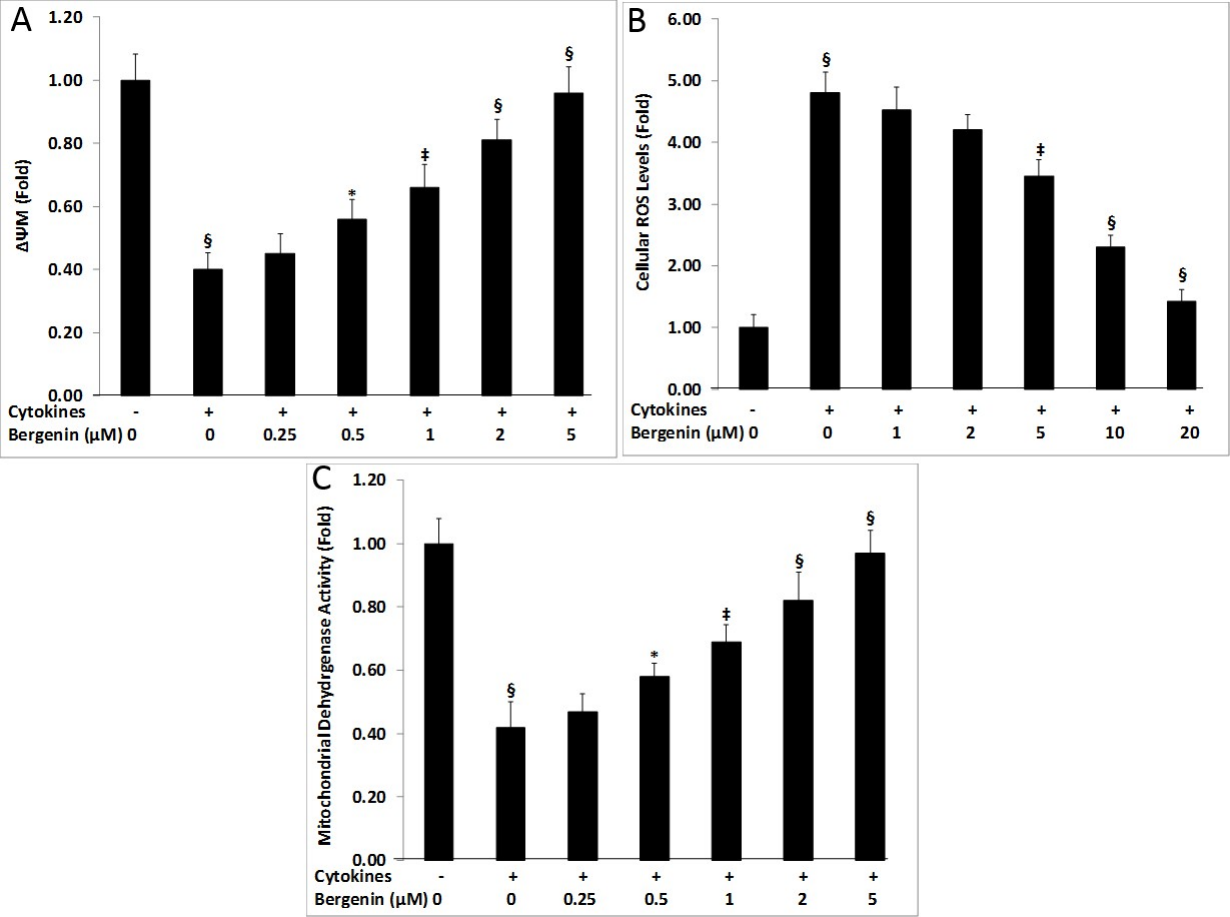
Cellular effects of bergenin on mitochondrial physiological parameters of beta cells in the presence of cytokines. Effects of bergenin on mitochondrial membrane potential (ΔΨm), intracellular ROS production, and mitochondrial dehydrogenase activity (MDA) after 48 hr treatment with IL-1β, INF-γ, and TNF-α. For dose-response studies, INS-1E cells were treated with various concentrations of bergenin (0.25–10 μM). The treated INS-1E cells were assessed for their ability to restore mitochondrial membrane potential (ΔΨm) (A); to reduce cellular ROS production (B); and to improve mitochondrial dehydrogenase activity (C). Data are represented as the mean ± standard deviation of 12 independent wells for A-F. * indicates p <0.05, ‡<0.01, and §<0.001 relative to cytokine-treated cells.

Apoptotic cells manifest a loss of mitochondrial membrane potential (ΔΨm). Tow-day treatment of INS-1E cells with a cytokine cocktail reduced mitochondrial membrane potential to almost 2.5-fold as compared to the untreated controls (Fig 3A); however, the addition of bergenin completely restored mitochondrial membrane potential in a dose-dependent manner. Beta cell mitochondrial membrane potential was increased to ˃90% of untreated levels by 5 μM bergenin (Fig 3A).

### Inhibition of the cytokine-induced ROS production by bergenin

Proinflammatory cytokines are reported to induce ROS production, which ultimately lead to beta cell dysfunction and death. Two-day treatment of INS-1E cells with a cytokine cocktail resulted in ˃4.5-fold increase in the ROS levels as compared to the untreated controls (Fig 3B); however, bergenin was found to be effective in reducing cellular ROS production in a dose-dependent manner. Beta cells ROS levels was found to be reduced to ˃60% by 10 μM bergenin (Fig 3B).

### Bergenin improves mitochondrial dehydrogenase activity

Inflammatory cytokines also known to impair mitochondrial physiology, disrupting electron transport chain, leading to beta cell dysfunction. Two-day treatment of INS-1E cells with a cytokine cocktail resulted in ˃2-fold decrease in the mitochondrial dehydrogenase activity as compared to the untreated controls (Fig 3C); however, addition of bergenin completely restored mitochondrial dehydrogenase activity in a dose-dependent manner. Beta cell mitochondrial dehydrogenase activity was increased to ˃80% of untreated levels by 2 μM bergenin (Fig 3C).

### Analysis of beta cells apoptosis using Flow Cytometry

**Fig 4:**
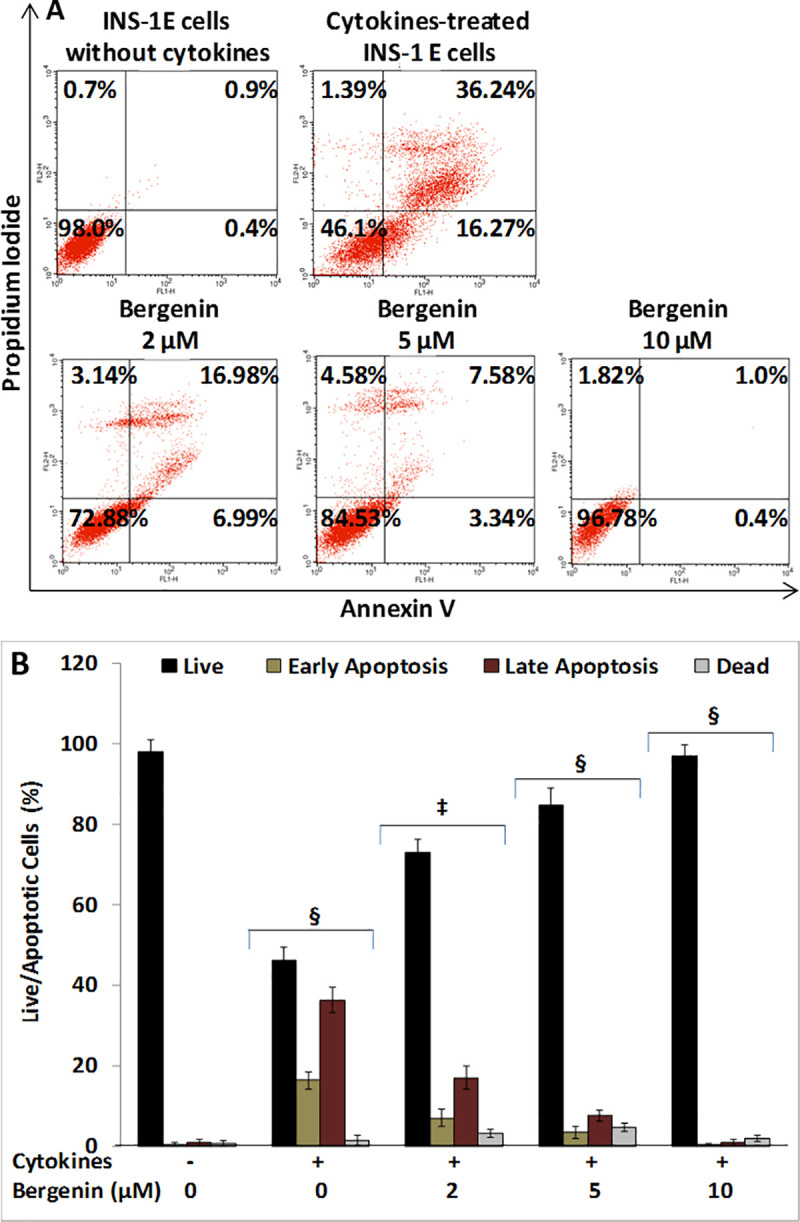
Bergenin suppresses cytokine-induced apoptosis in beta cells. INS-1E cells were treated with cytokines (IL-1β, INF-γ, and TNF-α) in the presence or absence bergenin (2–10 μM) for 48 hr, and were assessed for apoptosis inhibition. (A) Represents the flow cytometric analysis of INS-1E cells treated with bergenin in the presence of cytokines cocktail. The viable cell populations are in the lower left quadrant (Annexin V^-^/PI^-^); the cells at the early apoptosis are in the lower right quadrant (Annexin V^+^/PI^-^); and the ones at the late apoptosis are in the upper right quadrant (Annexin V^+^/PI^+^); and dead cells are in the upper left quadrant (Annexin V^-^/PI^+^). (B) Represents the live and apoptotic beta cell population after treatment with cytokines and bergenin. Data are represented the mean ± standard deviation of 2 independent experiments. * indicates p <0.05, ‡<0.01 and § <0.001 relative to cytokine-treated cells.

Using Flow Cytometry, we also examined the effects of bergenin on the suppression of cytokine-induced beta cell apoptosis. Two-day treatment of INS-1E cells with a cytokine cocktail caused approximately 50% of beta cell apoptosis when compared to the untreated controls (Fig 4A and 4B); however, addition of bergenin significantly inhibited cytokine-induced beta cell apoptosis in a dose-dependent manner. Beta cell apoptosis was almost completely suppressed by 10 μM bergenin (Fig 4A and 4B).

## Discussion

**Fig 5:**
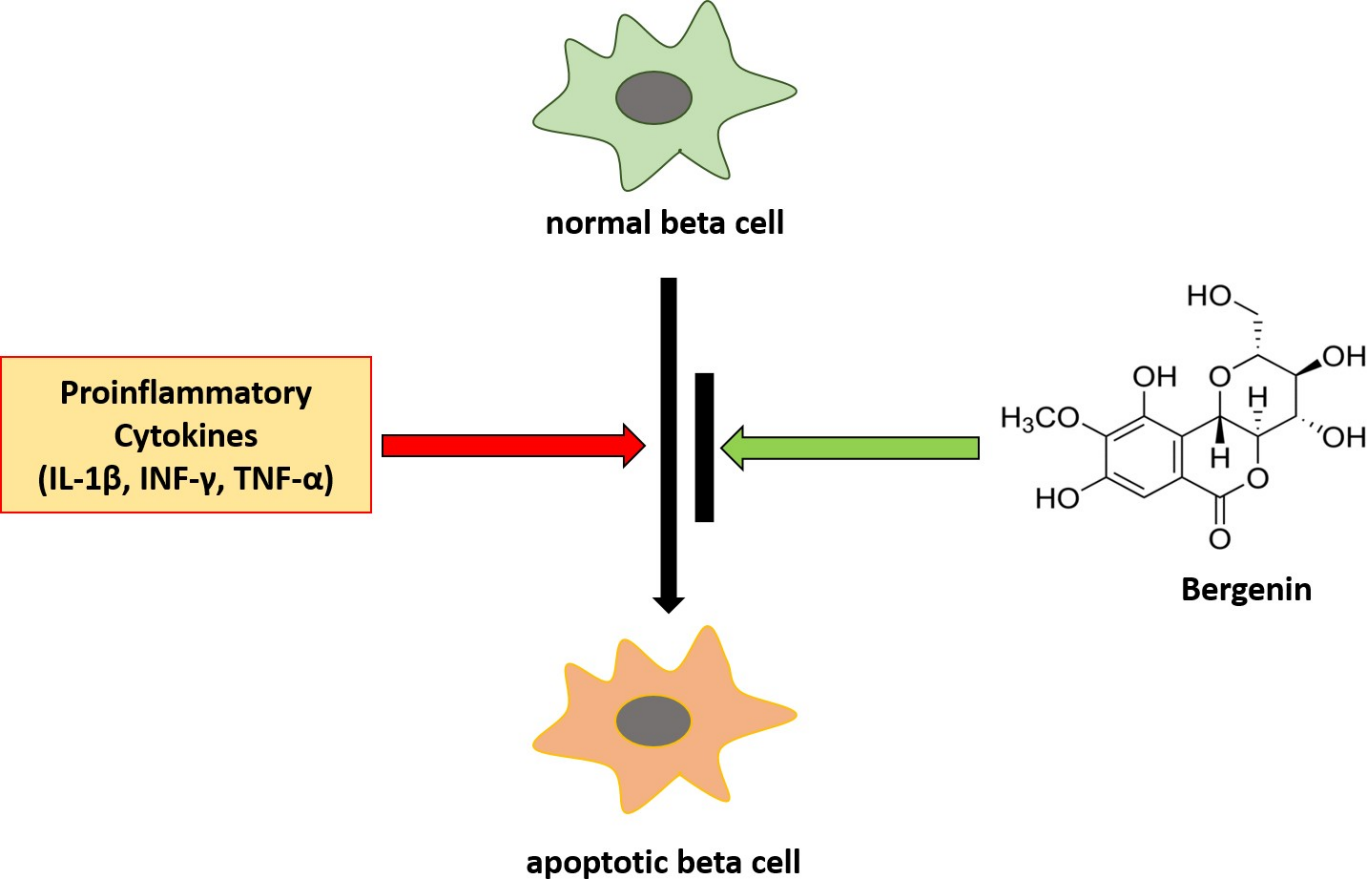
Bergenin suppressed cytokine-induced beta cell apoptosis. Exposure of beta cell to proinflammatory cytokines (IL-1β, INF-γ, and TNF-α) lead to apoptosis. However, the addition of bergenin suppressed deleterious effects of cytokine cocktail and prevented apoptosis in INS-1E cells.

In this study, we demonstrate for the first time that bergenin can inhibit beta cell apoptosis in the presence of cytokines (IL-1β, IFN-γ, and TNF-α) and concurrently increased beta cell viability and function (Fig 5). Bergenin exhibited potent pharmacological activity and significantly improved i) beta cell viability, ii) restored glucose-stimulated insulin secretion, iii) and improved mitochondrial physiology in INS-1E cells. Bergenin protected beta cells through multiple mechanisms simultaneously. Therefore, it could be of great clinical value for the prevention and treatment of diabetes.

Proinflammtory cytokines are reported to infiltrate pancreatic islets at early stage of diabetes leading to beta cell dysfunction and death [20, 28–30]. In beta cells, IL-1β, INF-γ, and TNF-α simulate intrinsic apoptotic pathway by inducing NFκB, MAPK, and STAT-1 signaling cascades which eventually cause reduction in beta cell mass [21, 23, 31, 32]. Since these cytokines play prominent role in beta cell biology, efforts have been made to identify small molecule suppressors which can prevent cytokine-induced beta cell death. Several studies have identified small molecule inhibitors of cytokine-induced beta cell apoptosis, such as histone deacetylases (HDACs) inhibitors, suberoylanilide hydroxamic acid (SAHA), and as trichostatin A (TSA) [33–36]. We have previously reported benzimidazole derivatives as suppressors of cytokine-induced beta cell death [25].

### Bergenin and beta cell viability

The rat insulinoma cell line INS-1E [37] was treated with a cytokine cocktail in the presence or absence of bergenin for 48 hr. We employed luciferase-based assay to evaluate the cellular ATP levels, which served as a surrogate marker for beta cell viability [38]. Bergenin significantly increased the cellular ATP levels in the presence of cytokines with an EC_50_ value of 1.97 μM (Table 1). Bergenin demonstrated antiarthritic activity through inhibition of the inflammatory cytokines (IFN-γ, TNF-α, and IL-2) in balb/c mice [39]. The alcoholic extracts of *Bergenia* rhizome exhibited anti-inflammatory effects in *in vivo* rat models [40, 41]. As bergenin being the main components of alcoholic and methanolic extract of *Bergenia* rhizome, we could speculate that bergenin could be responsible, at least in part, for the effects observed with these extracts. Furthermore, bergenin was also reported for anti-inflammatory and antinociceptive activities, which were due to the inhibition of IL-1β and TNF-α [42, 43].

**Table 1:** Activity profile of bergenin against beta cell viability, mitochondrial physiological parameters, beta cell function and apoptosis.

Cell-based Assays	EC_50_ (μM)	Max. Act. (%)	IC_50_ (μM)	Max. Act. (%)
**Cellular ATP Levels**	**1.97±0.47**	**95**	**-**	**-**
**Caspase-3 Activity**	**-**	**-**	**7.29±2.45**	**82**
**Cellular Nitrite Production**	**-**	**-**	**6.82±2.83**	**85**
**Mitochondrial Membrane Potential**	**2.27±0.83**	**95**	**-**	**-**
**Cellular ROS Production**	**-**	**-**	**14.63±3.18**	**71**
**Mitochondrial Dehydrogenase Activity**	**1.39±0.62**	**97**	**-**	**-**
**Glucose-stimulated Insulin Secretion**	**6.73±2.15**	**83**	**-**	**-**
**ATP in the Absence of Cytokines**	**-**	**IA**	**-**	**-**
**No Cytokines Cytotoxicity Assay**	**-**	**-**	**-**	**IA**

### Bergenin and beta cell apoptosis

Bergenin was further examined for its effects on different aspects of beta cell biology. We assessed inhibition of caspse-3 activity as a direct indicator of apoptosis. Generally, caspase activity is highly increased in apoptotic cells. Similarly, two-day treatment of INS-1E cells with a cytokine cocktail increased caspase-3 activity; however, this increase in caspase activity was significantly suppressed by the addition of bergenin to the INS-1E cells with an IC_50_ value of 7.29 μM (Table 1). This observation demonstrates for the first time a protective and or beneficial role of bergenin in suppressing beta cell apoptosis. We were also able to demonstrate inhibition of the cytokine-induced beta cell apoptosis through flow cytometry analysis.

### Bergenin and nitric oxide

IL-1β, and IFN-γ, are reported to stimulate the expression of inducible nitric oxide synthase (iNOS), which causes NO formation leading to beta cell death. Using Griess reagent, we assessed cellular nitrite production, a surrogate marker for NO. Bergenin has been reported to downregulate the expression of inducible nitric oxide synthase (iNOS), thereby reducing NO production [44, 45]. Similarly, in our current study, the cytokine cocktail we used highly elevated NO production in INS-1E cells; however, addition of beregnin significantly reduced NO levels in beta cells with an IC_50_ value of 6.82 μM (Table 1).

### Bergenin and mitochondrial physiology

Mitochondrial physiology is also impaired by the adverse effects of cytokines in beta cells. Two-day treatment of INS-1E cells with inflammatory cytokines reduced mitochondrial membrane potential, which was completely restored by the addition of beregnin with an EC_50_ value of 2.27 μM (Table 1). Generally, cellular ROS are in dynamic equilibrium, but increased ROS production due to inflammatory factors, may induce NF-κB, JAK/STAT, and MAPK pathways resulting in beta cell apoptosis [46]. Bergenin contains five hydoxyl groups, which play important role in its antioxidant activity. Several studies have reported anioxidant potential of bergenin in the context of lipid peroxidation, hydrogen peroxide, DPPH, and ABTS scavanging assays [47–49]. In our current study, two-day treatment of INS-1E cells with a cytokine cocktail increased ROS production, which was effectively reduced by bergenin with an IC_50_ value of 14.63 μM (Table 1). Mitochondrial membrane potential was also impaired in beta cells by the exposure to the cytokine cocktail; however, addition of bergenin almost completely restored membrane potential with an EC_50_ value of 2.27 μM (Table 1). Moreover, mitochondrial dehydrogenase activity was reduced by the adverse effects of cytokine cocktail in INS-1E cells, was completely restored by bergenin with an EC_50_ value of 1.39 μM (Table 1). These effects of bergenin in improving mitochondrial physiology in INS-1E cells in the presence of cytokines are reported for the first time.

### Bergenin and insulin secretion

The main physiological function of beta cells is to secrete insulin following glucose exposure. In the current study, two-day treatment of INS-1E cells with a cytokine cocktail reduced glucose-stimulated insulin secretion; however, addition of bergenin significantly restored insulin secretion in beta cells with an EC_50_ value of 6.73 μM (Table 1). These data suggest the beneficial role of bergenin on beta cell insulin secretory function.

**Fig 6:**
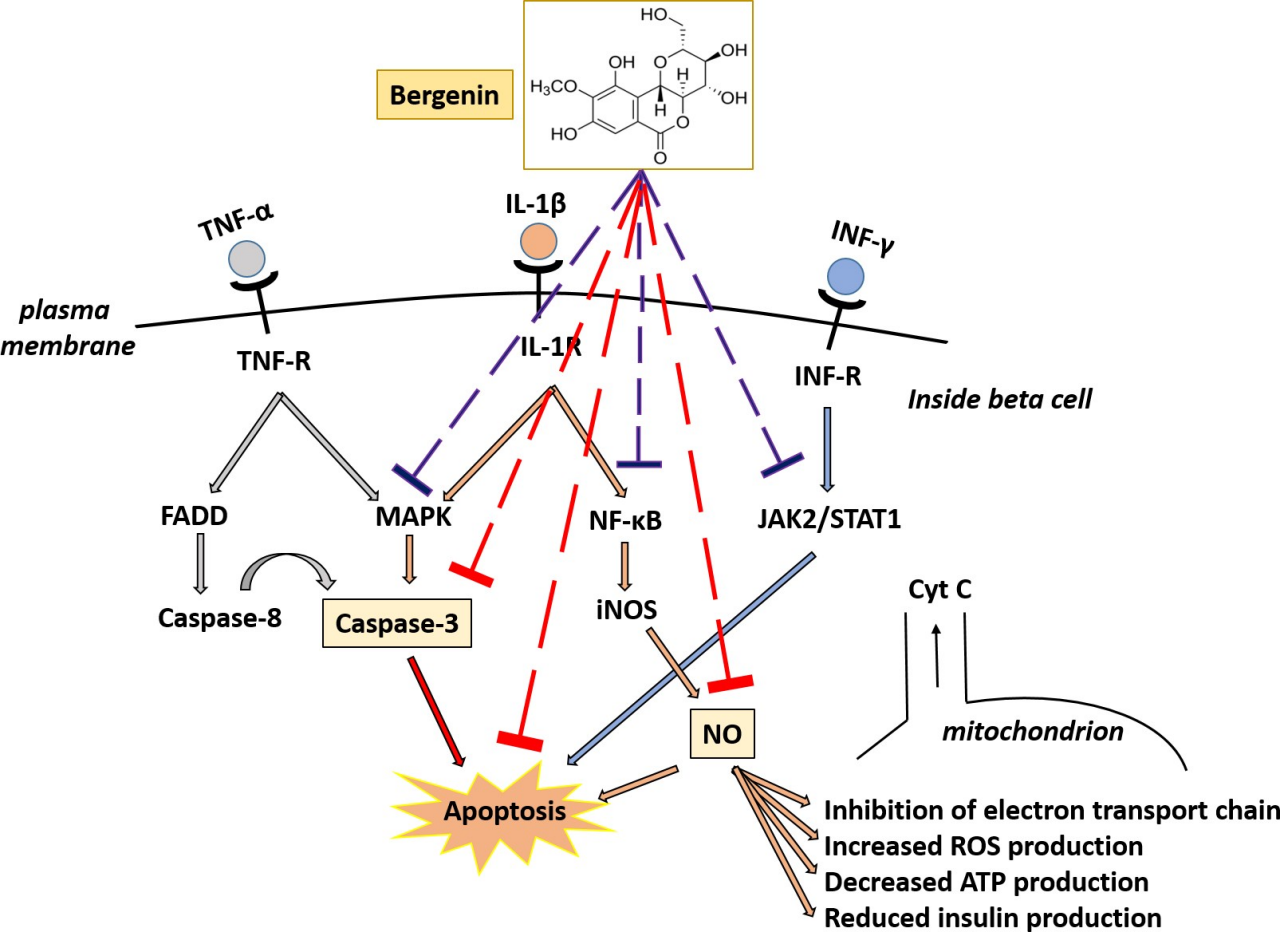
Potential mechanism of action of bergenin in suppressing cytokine-induced apoptosis in INS-1E cells. Proinflammatory cytokines (IL-1β, INF-γ, and TNF-α) initiate a cascade of signaling pathways leading to beta cell apoptosis. These cytokines stimulate JAK-STAT, NFκB, and MAPK pathways, which later induce intrinsic apoptotic pathway in beta cells. IL-1β also induces nitric oxide (NO) production, which causes inhibition of electron transport chain, increase in ROS production, decrease in glucose oxidation rate resulting in reduced ATP generation, and insulin production. Bergenin protected pancreatic beta cells through multiple mechanisms simultaneously. Bergenin suppressed beta cell apoptosis by potentially influencing NF-κB, MAPK and JAK/STAT pathways (represented by blue dashed lines). This is evident from the inhibition of downstream effector molecules (caspse-3, NO, ROS, and apoptosis) targeted in cell-based assays (represented by red dashed lines). By inhibiting cytokine-induced NO production, bergenin was able to increase in cellular ATP levels, decreased ROS production, and increased insulin production.

In the present study, bergenin exhibited multiple pharmacological activities simultaneously while protecting beta cells from the deleterious effects of inflammatory cytokine (Fig 6). Bergenin seems to be potentially influencing NF-κB, MAPK, and JAK/STAT pathways, as evident from the inhibition of downstream effector molecules of these pathways, such as caspse-3, NO, ROS, and apoptosis. By inhibiting cytokine-induced NO production, bergenin was able to increase ATP levels, decrease ROS production, and increase insulin production.

## Conclusion

The present study shows for the first time that bergenin could be a potentially interesting candidate for the prevention and or treatment of diabetes. In INS-1E cells, bergenin increased beta cell viability, suppressed cytokine-induced beta cell apoptosis, and restored beta cell insulin secretory function. Bergenin protected beta cells through multiple mechanisms simultaneously. The anti-apoptotic effect of bergenin could be due the reduction in caspase-3 activity, NO and ROS production by potentially influencing NF-κB, MAPK, and JAK/STAT pathways. Therefore, it is of great clinical value for the prevention and treatment of diabetes. Due to the complexity of natural products and their mechanisms, the exact targets and mechanisms need to be further explored and verified in isolated pancreatic islets or in animal models of diabetes.
